# Editorial: Vascular Factors and Vascular Lesions in Pre-clinical Alzheimer's Disease

**DOI:** 10.3389/fneur.2021.738465

**Published:** 2021-09-01

**Authors:** Michael Malek-Ahmadi, Yi Su, Willemijn J. Jansen

**Affiliations:** ^1^Banner Alzheimer's Institute, Phoenix, AZ, United States; ^2^Department of Psychiatry and Neuropsychology, Alzheimer Center Limburg, School for Mental Health and Neuroscience, Maastricht University, Maastricht, Netherlands

**Keywords:** APOE, cardiovascular disease, cerebrovascular disease, dementia–Alzheimer disease, mild cognitive impairment

The role of vascular factors (VFs) in Alzheimer's disease (AD) continues to generate significant discussion and debate. Neuropathological studies have consistently shown that AD and cerebrovascular neuropathologies have a high rate of co-occurrence in older adults (1, 2), even in those who are cognitively unimpaired (CU) at time of death (3). Much of the debate regarding the confluence of AD and VFs has centered around the degree to which the two might be mechanistically related, as this will determine the clinical expression and prognosis of AD. Although there is evidence suggesting that VFs are independent of AD pathways (4, 5), there is increasing evidence suggesting a synergistic relationship (6–8). The latter is supported by studies demonstrating that AD and cardiovascular disease share a common genetic risk factor in APOE ε4 (9, 10).

Aside from common VFs, such as high blood pressure and high cholesterol, there is increasing interest in blood-based and cerebrovascular markers that may provide greater insight into specific vascular mechanisms that may play a part in AD pathogenesis. Results from a meta-analysis of genome-wide association studies indicate that there are three distinct cardiovascular-related pathways to AD (11) while several other studies have shown that the presence of certain cardiovascular-related gene polymorphisms may contribute to increases in both AD risk and pathology (12, 13). While these lines of evidence still require further validation, they serve as the foundation for the rationale of this Research Topic which aims to continue the on-going work dedicated toward elucidating the role that VFs may have in AD.

In this Research Topic we present a series of articles that provide new insights and perspectives into the relationship between VFs and AD. Kim et al. provide a review on the of role of cerebral small vessel disease in AD. Specifically, their discussion of findings showing that white matter hyperintensities are associated with both cognitive decline and tau pathology demonstrate that both the clinical and neuropathological presentation of AD are impacted by VFs. Kim et al. discussion of genetic factors also highlights the possible confluence of vascular-related genes and APOE ε4, but at the same time demonstrates the need for AD researchers to give greater consideration to non-APOE genetic factors that may affect downstream pathological and clinical changes. Finally, their discussion of possible mechanisms that underlie the relationship between cerebrovascular changes and AD pathology highlight the roles of cerebral hypoperfusion and blood-brain barrier disruption as factors that lead to the accumulation of AD pathology. While these associations are still being validated in experimental studies, the summary of findings by Kim et al. help set the stage for this Research Topic.

Raghavan et al. provide an interesting study that investigates factors that underlie differences between silent brain infarctions and symptomatic brain infarctions where they report that hypertension and alcoholism were associated with a more than 4-fold increase in the odds of having silent brain infarctions. In addition, they report on laterality and location differences between silent and symptomatic brain infarctions. Sim et al. report on the associations between hippocampal enlarged perivascular spaces (H-EPS), medial temporal lobe atrophy (MTA), and cognition. Their primary finding was that cognition was not associated with H-EPS, although hippocampal volume and H-EPS showed a small, but significant association. They propose that H-EPS may be a secondary consequence of hippocampal neurodegeneration. An additional finding is that H-EPS did not correlate with cerebrovascular measures and that H-EPS is likely the result of normative aging effects.

Finally, Keage et al. report on the use of event-related potentials (ERPs) associations with cardiometabolic burden in older adults. This study used an n-back working memory task during EEG acquisition to evoke P1, N1, and P3b signals and found that cognitive performance did not differ by levels of cardiometabolic burden, but that P3b ERPs showed significant inverse associations with cardiometabolic burden. Keage et al. suggest that P3b changes may be a more sensitive measure of cardiovascular-related brain changes than cognitive measures and they propose that P3b changes could be used in lieu of cognitive measure to assess brain function in cardiovascular intervention studies. In addition, they highlight the possibility that ERPs may be more sensitive to intervention-related functional brain changes than cognitive tests. Another interesting suggestion these authors put forth is that the possible confounding effects of cardiometabolic group differences should be considered in future ERP studies.

While the relationship between VFs and AD continues to be of great interest across the AD research spectrum, there is a need to broaden the perspective by which these associations are assessed. The studies included in this Research Topic provide novel and interesting perspectives by which the role of VFs in AD may be elucidated, both in terms of pathogenic factors and empirically-based measures of brain function.

## Author Contributions

All authors listed have made a substantial, direct and intellectual contribution to the work, and approved it for publication.

## Conflict of Interest

The authors declare that the research was conducted in the absence of any commercial or financial relationships that could be construed as a potential conflict of interest.

## Publisher's Note

All claims expressed in this article are solely those of the authors and do not necessarily represent those of their affiliated organizations, or those of the publisher, the editors and the reviewers. Any product that may be evaluated in this article, or claim that may be made by its manufacturer, is not guaranteed or endorsed by the publisher.
